# Human epigenetics and microbiome: the potential for a revolution in both research areas by integrative studies

**DOI:** 10.4155/fsoa-2017-0046

**Published:** 2017-06-09

**Authors:** Franck Carbonero

**Affiliations:** 1 Department of Food Science, University of Arkansas, Fayetteville, USA

**Keywords:** epigenetics, health and disease, microbiome

A simple PubMed search confirms the intuitive thinking that human epigenetics and human microbiome research have received considerable attention in the recent years (13,082 and 28,446 hits, respectively). It is therefore astonishing that the two fields are very rarely studied together (118 results for ‘human epigenetics microbiome’ search, but only 3 actual research articles among literature reviews and opinions articles), while the need for interdisciplinary studies is often called for. It makes perfect sense that the host-associated microbiome may be the ultimate environmental trigger for epigenetic processes, because of its spatial location and ability to convert environmental and diet-derived compounds before they reach human cells. It is already known that the dynamic nature of human epigenetics is a significant hurdle for clinical translation of basic research, and the human microbiome, which is both personalized and dynamic, is probably an additional significant confounder.

## So why try to correlate epigenetics & microbiome?

First, as we reported last year [1], the extent to which epigenetic mechanisms modulate healthy or commensal microbes is virtually unknown, let alone how those processes are influenced and what influence they can have on their surrounding environment. This means that potentially any association with health and disease that have been suggested in the recent years [2,3] may have to be revisited through the microbiome epigenome perspective [4]. Understandably, investigators in microbiome research tend to consider microbes’ metabolic properties as relatively stable, as illustrated by the popular use of bioinformatics algorithms (Phylogenetic Investigation of Communities by Reconstruction of Unobserved States [PICRUST]) that supposedly infer metabolic properties based on taxonomic (based on phylogenetic markers) profiles [5]. While the inherent limitations of such an approach are generally well discussed, the fact that epigenetic regulation of gene expression in the vast majority of human microbiome members is unknown represents another major limitation that has so far been ignored. Second, human microbiome is remarkably personalized [6]; thereby its influence on host epigenetic processes will be too. To put it simply into an ideal model, if two completely identical organisms were to be subjected to the exact same environmental conditions, their epigenetic regulation could still be divergent because of different metabolic activities of their personal microbiomes. Finally, there is still little understanding on how human microbiome modulates host epigenetic processes directly or indirectly. And the few popular paradigms, such as the heavily cited histone deacetylation in human colonocytes by microbially derived butyrate [7], probably still need to be refined.

## Challenges & potential directions for integrative human microbiome & epigenetic research

Now, combining human epigenetic and microbiome research is surely easier said than done. While the most commonly used molecular methods (e.g., high-throughput sequencing, PCR and associated techniques) clearly overlap between the fields [8–10], data analysis and interpretation are already significantly different. However, the critical point is how to choose appropriate models and sample types, and I will focus on the digestive tract to illustrate the methodological issues. While overwhelmingly dominant in gut microbiome studies, stool samples are well known to be somewhat inappropriate representation of the actual gut microbiome. Biopsies, on the other hand, are better proxy of the microbial ecosystems surrounding epithelial cells, but not of the overall colonic ecosystem (not to mention that their sampling is inherently restricted to medical needs). Subsequently, it would appear that a more realistic approach would be to first determine baseline knowledge from different models.

From the microbiome members’ perspective, there is already some knowledge about genomic fine-tuning in the model human gut symbiont *Escherichia coli* due to environmental conditions (inside vs outside digestive tract) [11,12]. It is very likely that epigenetics actually play an even greater role into maximizing microbial populations’ fitness to the ever-changing gut (micro)ecosystems. Therefore, there is a need to determine the amount of potential epigenetic control on (initially) abundant gut microbiome members. One recent report can be used as a blueprint for such projects. Leonard *et al*. performed a full methylome analysis of two strains of *Bacteroides dorei* isolated from two different stool samples [4], and found an outstanding difference in the number of methylation sites present in the two virtually similar genomes. To obtain a preliminary view on methylation potential, one approach would be to obtain methylome from a large collection of isolates obtained from a single stool sample. From such a baseline, it would then become possible to start hypothesis-based studies.

While linking host epigenetic profiles with gut microbiome profiles has been attempted in a few previous studies [13,14], this kind of approach should probably be limited to cases where a relatively clear-cut segregation power has been identified in either the epigenetic or the microbiome profile. And even in those cases, the dynamic nature of the microbiome (particularly, in the gut) could become a strong confounder, as for example, stool sampling events cannot be standardized and scheduled to fit the scientific objectives of research investigators. Another option we are currently attempting in the swine model is to directly sample adjacent luminal content for gut microbiome analyses and colonic mucosa for epigenetic profiling. While this approach should arguably reduce confounding factors, it would have to be primarily applied to animal models, since human colonic biopsy sampling is relatively uncommon, and may not even allow for studying both microbiome and host epigenetics.

There has also been extensive research into chemical compounds that could serve as epigenetic drugs [15]. In that context, metabolomics, the study of all metabolites resulting from microbial metabolism found across the human body [16], should definitely be considered more often. Indeed, molecules of interest may readily be produced *in situ* by specific microbes; and molecules of interest may actually be degraded or inactivated by microbial activity, as shown previously for a popular cardiac disease drug [17]. In addition, the metabolome clearly represents another parameter shaping epithelial cells surrounding environment. Since gut microbiome and gut and urine metabolome appear to correlate well, enough that the urine metabolome can be used as a proxy [18], it is possible that stool and/or urine would actually be the appropriate parameter to combine with host epigenetics profiling-based studies.

## Conclusion

To summarize, there is interest and rationale to consider the human microbiome as a novel and crucial parameter in clinical epigenetic research. It is rather evident that attempting to perform large exploratory studies may be too ambitious based on our current knowledge. A reductionist approach is advised to explore both epigenetic controlling of the human microbiome, and how human microbiome and metabolome can modulate human epigenetic regulation.
